# Remedies for Cancer

**Published:** 1897-04-17

**Authors:** G. Munro Smith

**Affiliations:** Senior Surgeon to Out-patient Department Bristol Royal Infirmary


### "REMEDIES FOR CANCER."

By G. Munro Smith, L.R.C.P.Lond., M.R.C.S., Senior Surgeon to Out-patient Department Bristol Royal Infirmary.

Is cancer ever curable except by the knife? A few years ago an answer in the affirmative would brand one as a quack; and, even now, the majority of surgeons would look with some distrust upon anyone who attached any real importance to the cases occasionally reported of amelioration by internal remedies. An attitude of scepticism on this subject is, no doubt, desirable, for so terrible a disease is bound to produce hundreds of so-called "cures," which are utterly worthless, but which are eagerly clutched at by patients and their friends. Nevertheless, it is one's duty to now and then reopen the subject, and hear what can be said on the other side. And, firstly, does spontaneous cure ever take place? Mr. Pearce Gould reported a case at the Clinical Society of London last November, of a woman, aged forty-three, who was admitted into the Middlesex Hospital with recurrent scirrhus of the breast. Small hard tubercles appeared in and near the scar of the operation, and large masses of glands existed in the axilla and above the clavicle. Nothing was done, yet these growths gradually dwindled, and ultimately completely disappeared. This is by no means a solitary experience; a sufficient number of somewhat similar instances have been recorded to enable us to say, without much hesitation, that the spontaneous cure of cancer actually does take place; and this enables us to accept rather more easily some of the reported effects of certain remedies. Of course, the all-powerful Roentgen rays have been called in to our aid. M. Despeignes[1] narrates the case of a patient, with all the symptoms of malignant disease of the stomach, who was much improved by subjecting the abdomen to these rays. The pain lessened, and the obvious swelling decreased in size.

In another case of cancer of the stomach, Brissaud[2] obtained good results by giving large doses (12 to 16 grammes daily) of chlorate of sodium; and M. E. Duvrac[3] got some improvement by the same means in a case of advanced uterine cancer; but he looks upon it only as a palliative treatment, not as a cure. Iodide of potassium frequently causes a slight temporary improvement in epithelioma of the tongue, and in cases where there is some doubt as to the diagnosis between this and syphilis, this amelioration has increased the difficulty of coming to a decision.

One of the most interesting drugs in this connection is that made from the juice of the celandine (Chelidonium majus), which formerly had a great repute as a corn cure. Dr. Denisenko, of Brjansk, has obtained very striking results in several cases. He applies a fluid extract to the part, and injects it subcutaneously as well as giving it internally. Dr. Samson visited Dr. Denisenko in Russia, and was convinced of the utility of this drug. He saw thirty patients under treatment.[4] Others have tried this without obtaining any good results.

The method of serum therapy has been carried out by Richet and Héricourt. Their plan is to triturate freshly removed carcinomatous or sarcomatous tissue with water, and inject the fluid, after filtering, into dogs. The animals' serum is used. In six cases of cancer treated in this way two received no benefit, three a temporary amelioration, whilst one was cured. This last was a case of malignant disease of the rectum in a woman, and the diagnosis is rendered rather uncertain by the fact of a distinct history of syphilis, and it does not appear that a sufficient trial was given to iodide of potassium or mercury.

M. Dubois[5] reports three cases treated in this way, two of which were very much improved. Other cases have been published. It has been suggested that some of the good results were due to inflammatory changes brought about by the injections.

Another method which has met with occasional good results is the injection of erysipelas toxins. A case is reported by Mr. Haslam[6] of a malignant tumour of the breast, with enlarged axillary glands, treated in this way with complete recovery.

Arsenic, which sometimes causes the disappearance of enlarged lymphatic glands and otherwise modifies tissue change, has been given in sarcomata of the skin with encouraging results, but there is as yet very little direct evidence as to its action on malignant growths.

Thyroid extract, as a powerful promoter of absorption, might be expected to occasionally give results in tumours other than lymphatic or thyroid, and such appears to be the case. Thus, Dr. Beatson has reported in the *Lancet* a case of severe mammary cancer which was apparently cured by the administration of this substance; and Dr. Robert Bell narrated three cases of the epithelioma of the cervix which he considered were much improved by it.[7] It should be added, however, that these patients were also curetted, so that it is not quite clear how much of the improvement was due to the extract.

It should be noticed, too, that several of the reported cures by this and other methods were in women at or about the climacteric, when the metabolism undergoes profound changes.

But, making all necessary deductions for possible errors in diagnosis, &c., the amount of evidence is decidedly in favour of an occasional cure of malignant disease without operation, and, as a drowning man will "grasp at a straw," so must all those who have to deal with that terrible condition—cancer that has advanced beyond the reach of the knife—hail with delight any possible method of treatment that may improve the patient's condition. As before stated, our attitude must be sceptical; we must be careful not to excite expectations which would probably be disappointed; but, on the other hand, we may now hope where formerly there was only despair.

[1] Lyon Médicale, July 27, 1896. [2] Gaz. Méd. de Strasbourg, May 1st. [3] Semaine Médicale, July 15, 1896. [4] B. Medical Journal, Feb. 6, 1897. [5] Semaine Médicale, Aug. 19, 1896. [6] Med. Journal, Feb. 1, 1896. [7] British Gynæcological Society's Meeting on May 14th, 1896.

